# The impact of an intervention to increase follow-up blood cultures for patients with *Staphylococcus aureus* bacteriuria

**DOI:** 10.1017/ash.2025.10067

**Published:** 2025-07-17

**Authors:** Jared Olson, Vincent Anella, Brandon J. Webb, Andrew T. Pavia, Emily A. Thorell, Adam L. Hersh, Dustin Waters

**Affiliations:** 1 Division of Infectious Diseases, Department of Pediatrics, University of Utah, Salt Lake City, UT, USA; 2 Department of Pharmacy, Primary Children’s Hospital, Salt Lake City, UT, USA; 3 Division of Infectious Diseases, Intermountain Medical Center, Murray, UT, USA; 4 Department of Pharmacy, McKay Dee Hospital, Ogden, UT, USA

## Abstract

**Background:**

*Staphylococcus aureus* bacteriuria (SABU) may represent bacteremia in a subset of patients. We describe the impact of a microbiology alert recommending follow-up blood cultures (FUBC) for patients with SABU in a large integrated health system.

**Methods:**

We conducted a quasi-experimental implementation study in adult ambulatory patients with documented SABU. We excluded patients with confirmed SAB up to 14 days prior to index SABU culture and with blood cultures obtained on the day of SABU. The primary outcome was rate of FUBC (collected between 1 and 5 days of SABU) among all cases of SABU. Secondary outcomes included percentage of patients with early SAB (collected between 1 and 5 days of SABU). We used interrupted time series analysis to compare rates of FUBC pre vs postintervention.

**Results:**

A total of 2 540 patients were identified; 1 213 (48%) were male. By the end of the postintervention period, the rate of FUBC (20.6%) had increased by 6.3 percentage points (*P* = .005) compared to the counterfactual (14.2%) had no intervention taken place (44.5% relative increase). Early SAB detection due to FUBC increased from .6% preintervention to 2.0% postintervention (*P* = .004).

**Conclusion:**

The microbiology alert initiative increased FUBC in patients with SABU by 44%, but the overall rate of FUBC remained low. The intervention increased early SAB detection. Risk-targeted strategies are needed to optimize FUBC collection in patients with SABU.

**Key summary points**

Why carry out this study?

- *Staphylococcus aureus* bacteriuria (SABU) is associated with *Staphylococcus aureus* bacteremia (SAB).
- It is unclear if increasing follow up blood cultures (FUBC) in cases of SABU will increase early detection of SAB.

What was learned from the study?

- The intervention increased FUBCs and detection of SAB in patients with SABU.
- Risk-targeted strategies are needed to target FUBC toward patients who are at highest risk for SAB.

## Introduction

In the absence of bladder catheterization, instrumentation or surgery, *Staphylococcus aureus* is an uncommon urinary pathogen; however up to 4% of positive urine cultures isolate *S aureus*.^1–3^ These positive cultures may represent contamination, colonization, or rarely primary urinary tract infection. However, in some cases, *S aureus* bacteriuria (SABU) is a manifestation of *S. aureus* bacteremia (SAB). In fact, SAB may be present in up to 30% of patients with SABU, with a wide range of severity and symptoms.^2,4^ Concomitant SAB and SABU is associated with increased risk of in-hospital mortality compared to SAB alone and may indicate higher endovascular inoculum with spillover into the urinary tract.^5,6^

Increasing follow-up blood cultures (FUBC) in select patients with SABU might lead to increased SAB detection and provide opportunities for earlier treatment. SABU detected ≥48 hours before SAB has been associated with higher risk of death compared to simultaneous SAB and SABU.^7^ More timely identification of SAB in these patients might improve outcomes. Male sex, inpatient status, pure *S. aureus* growth, urinary procedures, and systemic signs of infection have been identified as risk factors for SAB in patients with SABU, but none is strongly discriminant. As a result, some researchers have recommended that at minimum, blood cultures should be obtained in patients with SABU and evidence of systemic inflammatory response.^4^ Microbiology comments accompanying culture results notifying providers of the potential that SABU may be an indicator of bacteremia have been shown to increase rates of FUBC.^7^

We hypothesized that deployment of clinician-targeted electronic decision support linked to *S. aureus*-positive urine cultures would increase screening rates and increase timely detection of SAB. Here we describe the impact of an electronic medical record embedded microbiology alert recommending FUBC in all patients with SABU in a large integrated health system.

## Methods

### Context

Intermountain Health (IH) is a fully integrated health system comprised of 34 hospitals and 400 clinics in 6 western U.S. states. This quality improvement project took place in the IH hospitals and clinics in Utah and Idaho. This study was deemed exempt and informed consent was waived by the Intermountain Health Institutional Review Board.

Safety events including incorrectly interpreting SABU as colonization when it was likely an indicator of invasive disease were reported anecdotally by antimicrobial stewardship staff during AS meetings. A continuing education lecture regarding the implications of SABU was given to systemwide pharmacists in July 2021 with special attention to Emergency Department (ED) culture call backs since positive urine cultures were reviewed daily in EDs, often by a dedicated ED pharmacist. ED pharmacists were encouraged to discuss SABU with a physician and to recommend evaluation for SAB. In October 2021, a high priority electronic alert for all hospital and ED SABU cases was deployed for ID pharmacists to review and resolve during business hours.

### Intervention

We developed an electronic intervention to prompt providers of the necessity to consider invasive infection as a cause of SABU. A comment was added to the microbiology report for all urine cultures positive for *S. aureus* regardless of collection location that read “Blood cultures and evaluating for systemic infection are recommended. *S. aureus* is not a common urinary pathogen and might represent seeding from bacteremia or instrumentation.” This electronic, clinician-targeted intervention was implemented with accompanying education targeted to pharmacists emphasizing the clinical importance of SAB evaluation for patients with SABU in February of 2023.

### Study of the intervention

We performed a quasi-experimental implementation study to determine the impact of the microbiology alert on the rate of FUBC and timeliness of SAB detection in patients with SABU.

Patients ≥18 years of age presenting to emergency department (ED) and ambulatory settings with documented SABU were identified from the electronic medical record between June 2019 and January 2024. We excluded patients with confirmed SAB up to 14 days prior to index SABU culture. We also excluded patients with blood cultures obtained within 24 hours of collection of positive urine culture, inferring that bacteremia was at least suspected at the time of urine culture obtainment as evidenced by blood culture collection. The analysis was limited to the first case of SABU per patient. The preintervention period was defined as June 2019 through January 2023 and the postintervention period as February 2023 through January 2024.

The following demographics and clinical variables were collected from the electronic medical record: sex, race, ethnicity, age, care location including ambulatory setting, admission type, urine collection method (catheterized, clean catch, unspecified), colony forming units of *S. aureus*, *S. aureus* methicillin susceptibility, blood cultures obtained between 14 days prior and up to 90 days post SABU identification, ED revisit and hospital readmission up to 90 days from SABU, and 90-day all-cause mortality. Chart review of SAB cases was performed and the following additional clinical variables were abstracted: presence of urinary symptoms at SABU identification, comorbidities (diabetes, intravenous drug use, admission at long -term care facility at time of SABU, presence of cardiac, spinal or joint hardware, renal transplant recipient), urogenital foreign body defined as urolithiasis/nephrolithiasis, urinary catheter or renal stent at time of SABU, and identified source of bacteremia.

### Objectives and endpoints

The primary objective of the intervention was to increase FUBC in patients with SABU. The primary end point, FUBC, was defined as a blood culture obtained between 1 and 5 days after the index SABU culture. This range was chosen to allow for urine cultures to become positive (1–2 d) and for clinics or EDs to then coordinate patient notification and collection of FUBC. We hypothesized that increasing FUBC would lead to both increased detection of SAB and earlier identification of SAB cases. We measured the following secondary endpoints: overall SAB rate, and the rate of early SAB, which we defined as a positive blood culture for *S. aureus* between 1 and 5 days relative to late SAB, defined by positive culture between 6 and 90 days after the index SABU.

We recognized that while providers would ideally arrange FUBCs through laboratory visits, it was possible that the intervention might drive higher rates of early ED revisits for FUBC collection. We hypothesized that the intervention would not impact overall rates of 90-day readmissions, but that early detection of SAB might shift these admissions to an earlier time frame. To evaluate these potential secondary impacts of the intervention, we measured two healthcare utilization endpoints: ED revisits and hospital admissions within 90 days of SABU. We stratified these as early (SABU day + 1 to + 5) and late (+6 to + 90). We also reported 90-day all-cause mortality between time periods.

### Subgroup analysis by sex

Given male sex is a risk factor for SAB in the setting of SABU, a planned subgroup analysis evaluating the impact of the intervention by sex was conducted.

### Patients with SAB

For patients with identified SAB, we compared pre and postintervention rates of urinary symptoms at presentation, early identification of SAB, presence of deep-seated infections defined as epidural abscess, vertebral osteomyelitis, endocarditis, or presumed endocarditis, and 90-day mortality.

### Analysis

We summarized demographic and clinical characteristics comparing pre and postintervention groups. Continuous data were reported as median and interquartile range and categorical data were reported as number and proportion. For our primary analysis, we used interrupted time series (ITS) analysis using least squares regression to model rates of FUBC pre vs postintervention. We calculated three ITS effects: 1) the initial intercept change in rate of FUBC, which estimates the initial impact of the intervention as the difference between the observed rate and the counterfactual rate had no intervention taken place, 2) the difference in slopes between the preintervention and intervention period, which models the relative change in FUBC rate after the implementation, and 3) the absolute and relative difference between the observed and counterfactual rates of FUBC at the end of the intervention. We compared pre and postintervention groups using Wilcoxon signed-rank test for continuous data and for categorical data χ^2^ or Fisher’s Exact tests (for variables with cells <10). ITS was performed in Microsoft Excel and all other statistical analyses were performed using R statistical software package, version 4.2.3 (R Project for Statistical Computing).

## Results

### Patients

A total of 3 213 patients with SABU were identified between June 2019 and January 2024, and 2 540 met the inclusion criteria. Of these, 1996 were in the preintervention group and 544 were in the postintervention group. Eleven patients (.3%) were excluded due to SAB identification within 14 days before SABU. An additional 662 (20.6%) patients were excluded for having blood cultures collected within 24 hours of urine culture of which 335 (50.6%) were positive for SAB.

Baseline demographics were similar between treatment periods with slight differences in admission type (Table 1). Compared to the preintervention period, during the postintervention time there was better documentation of urine specimen collection method, lower CFUs of *S. aureus* bacteriuria, and higher rates of organism methicillin-susceptibility determinations.

**Table 1.** Demographics and outcomes by intervention period

Variable	Preintervention (*N* = 1996)	PostIntervention (*N* = 544)	*P*-value
Male, *n* (%)	968 (48.5%)	245 (45.0%)	.17
White, *n* (%)	1756 (88.0%)	483 (88.8%)	.66
Hispanic, *n* (%)	161 (8.1%)	49 (8.0%)	.66
Age, years (IQR)	63 (38,76)	62 (34,77)	.44
Urgent care, *n* (%)	386 (19.3%)	122 (22.4%)	.13
Emergency care, *n* (%)	399 (20.0%)	110 (20.2%)	.91
Urine Collection, *n* (%)			<.001
Catheterized	164 (8.2%)	68 (12.5)	
Clean Catch	976 (48.9%)	355 (65.3%)	
Unspecified	855 (42.8%)	121 (22.2%)	
Colony forming units/ml, *n* (%)			<.001
<50,000	389 (19.5%)	141 (25.9%)	
50,000–100,000	438 (21.9%)	133 (24.4%)	
>100,000	1 169 (58.6%)	270 (49.6%)	
Organism methicillin susceptibility, *n* (%)			.036
Methicillin susceptible *S aureus*	1 371 (68.7%)	392 (72.1%)	
Methicillin resistant *S aureus*	485 (24.3%)	130 (23.9%)	
Undifferentiated	140 (7.0%)	22 (4.0%)	
Antibiotic prescribed at urine culture collection, *n* (%)	936 (46.9%)	247 (45.4%)	.57
Oral cephalosporin	385 (19.3%)	99 (18.2%)	.61
Trimethoprim/Sulfamethoxazole	211 (10.6%)	55 (10.1%)	.82
Fluoroquinolone	169 (8.5%)	26 (4.8%)	.005
Nitrofurantoin	156 (7.8%)	59 (10.8%)	.03
Follow up blood culture, *n* (%)	177 (8.9%)	112 (20.6%)	<.001
*Staphylococcus aureus* bacteremia (SAB) within 3 months, *n* (%)	33 (1.7%)	14 (2.6%)	.22
Early *S. aureus* bacteremia, *n* (%)	12 (.6%)	11 (2.0%)	.004
Late *S. aureus* bacteremia, *n* (%)	21 (1.1%)	3 (.6%)	.41
Emergency department (ED) visit within 3 months, *n* (%)	378 (18.9%)	151 (27.8%)	<.001
Early ED revisit, *n* (%)	131 (6.6%)	70 (12.9%)	<.001
Late ED revisit, *n* (%)	247 (12.4%)	81 (14.9%)	.14
Readmission within 3 months, *n* (%)	219 (11.0%)	66 (12%)	.49
Early readmission, *n* (%)	54 (2.7%)	19 (3.5%)	.41
Late readmission, *n* (%)	165 (8.3%)	47 (6.8%)	.85

### Primary endpoint

**Figure 1.**
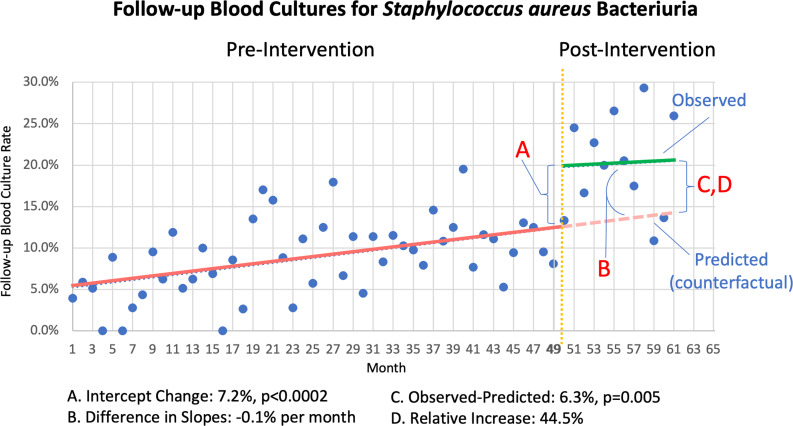
Interrupted time series analysis of follow up blood cultures for Staphylococcus bacteriuria.

Overall, 177 patients (8.9%) in the preintervention and 112 patients (20.6%) in the postintervention period had FUBC. In ITS analysis, the initial impact of the intervention was estimated as a 7.2% increase in FUBC relative to the counterfactual had no intervention taken place (*P* < .0002) (Figure 1). The difference in slopes (.1% *decrease* in FUBC per month) between the preintervention (.2% increase in FUBC per month) and postintervention period (.1% increase in FUBC per month) was not statistically significant. At the end of the study period, the observed rate of FUBC (20.6%) was greater than the counterfactual rate (14.2%) had no intervention taken place, corresponding to a 6.3% absolute increase in percentage of patients with FUBC (*P* = .005) and a 44.5% relative increase in FUBC.

### Secondary outcomes

Overall, SAB detection increased from 1.7% (*n* = 33) to 2.6% (*n* = 14) after the intervention (*P* = .22). Early SAB detection increased from .6% (*n* = 12) pre to 2.0% (*n* = 11) postintervention (*P* = .002) while late SAB detection decreased from 1.15% (*n* = 21) to .6% (*n* = 3; *P* = .41). The number of FUBC to detect one early SAB was 15 in the preintervention group and 11 in the postintervention group.

ED revisits within 90 days of SABU identification increased from 18.9% (*n* = 378) to 27.8% (*n* = 151) after the intervention (*P* < .001). This was driven by early ED revisits which occurred in 6.6% (*n* = 131) of patients in the pre and 12.9% (*n* = 70) in the postintervention period (*P* < .001). Late ED revisits increased from 12.4% (*n* = 247) to 14.9% (*n* = 81) after the intervention (*P* = .14).

Hospital readmissions within 90 days of SABU identification occurred in 11% (*n* = 219) of patients in the pre and 12% (*n* = 66) in the postintervention period (*P* = .49). Early readmissions occurred in 2.7% (*n* = 54) and 3.5% (*n* = 19; *P* = .41) and late readmissions occurred in 8.3% (*n* = 165) and 6.8% (*n* = 47; *P* = .85) of patients in the pre and postintervention periods, respectively.

All-cause 90-day mortality from SABU identification occurred in 4.4% (*n* = 87) of patients in the preintervention group and 4.4% (*n* = 24) of patients in the postintervention group (*P* = .96).

### Subgroup analysis by sex

The rates of FUBC were similar between males and females in the preintervention (9.3% vs 8.5%; *P* = .51) and postintervention periods (20.8% vs 20.4%; *P* = .91). Females with SABU differed in demographics and clinical characteristics including lower age, higher likelihood of urgent care urine evaluation, higher rates of clean catch specimens, and smaller number of colony forming units of *S. aureus* (Table 2). Despite similar rates of evaluation, FUBC were positive for SAB in 1 (.7%) of 148 females tested compared to 22 (15.6%) of 141 males tested (*P* < .001). Although 90-day ED revisits were similar between males and females (20.7% vs 20.9%; *P* = .91), 90 -day readmissions occurred more frequently in males than females (14.3% vs 8.4%; *P* < .001).

**Table 2.** Baseline demographics and outcomes by sex

Variable	Male (*N* = 1 213)	Female (*N* = 1 327)	*P*-value
White, *n* (%)	1 090 (89.9%)	1 149 (86.6%)	.013
Hispanic, *n* (%)	68 (5.6%)	142 (10.7%)	<.001
Age, years (IQR)	70 (57,79)	45 (27, 72)	<.001
Urgent Care, *n* (%)	193 (15.9%)	315 (23.7%)	<.001
Emergency Care, *n* (%)	256 (21.1%)	253 (19.1%)	.20
Urine Collection, *n* (%)			<.001
Catheterized	159 (13.1%)	73 (5.5%)	
Clean Catch	533 (43.9%)	798 (60.1%)	
Unspecified	520 (42.9%)	456 (34.4%)	
Colony forming units/ml, *n* (%)			<.001
<50,000	178 (14.7%)	352 (26.5%)	
50,000–100,000	215 (17.7%)	356 (26.8%)	
>100,000	820 (89.9%)	619 (46.6%)	
Organism methicillin susceptibility, n (%)			.001
Methicillin susceptible *S aureus*	797 (65.7%)	966 (72.8%)	
Methicillin resistant *S aureus*	329 (27.1%)	286 (21.6%)	
Undifferentiated	87 (7.2%)	75 (5.7%)	
Antibiotic prescribed at urine culture collection, *n* (%)	560 (46.2%)	623 (46.9%)	.72
Oral cephalosporin	211 (17.4%)	273 (20.6%)	.05
Trimethoprim/Sulfamethoxazole	169 (13.9%)	97 (7.3%)	<.001
Fluoroquinolone	134 (11.0%)	61 (4.6%)	<.001
Nitrofurantoin	34 (2.8%)	181 (13.6%)	<.001
Follow- up blood culture performed, *n* (%)	141 (11.6%)	148 (11.2%)	.76
Early *S aureus* bacteremia, *n* (%)	22 (1.8%)	1 (.1%)	<.001
Late *S aureus* bacteremia, *n* (%)	17 (1.4%)	7 (.5%)	.039
Emergency Department (ED) revisit within 3 months, *n* (%)	14 (20.7%)	278 (20.9%)	.91
Early ED revisit, *n* (%)	94 (7.7%)	107 (8.1%)	.83
Late ED revisit, *n* (%)	157 (12.9%)	171 (12.9%)	>.99
Readmission within 3 months, *n* (%)	174 (14.3%)	111 (8.4%)	<.001
Early readmission, *n* (%)	51 (4.2%)	22 (1.7%)	<.001
Late readmission, *n* (%)	123 (10.1%)	78 (6.7%)	.002

### Patients with SAB

During the study period, 47 patients with SAB were identified within 60 days of SABU. Urinary symptoms were common in patients with SAB and were present in 53.3% (16/33) of patients in the pre and 64.3% (9/14) in the postintervention group (*P* = .72). (Table 3) Early SAB was detected in 12 of 33 (36.4%) patients with SAB in the pre and in 11 of 14 (78.6%) in the postintervention group (*P* = .02) (Table 4). Deep seated infections were identified in 16 (48.5%) patients in the pre and 0 (0%) in the postintervention groups (*P* = .004). Mortality within 90 days occurred in 4 (12.1%) patients in the pre and 3 (21.4%) patients in the postintervention group (*P* = .71).

**Table 3.** Demographics for confirmed Staphylococcus aureus bacteremia by intervention period

Variable	Pre (*n* = 33)	Post (*n* = 14)	*P*-value
Male, *n* (%)	26 (78.8%)	13 (92.9%)	.45
White, *n* (%)	30 (90.9%)	13 (92.9%)	1
Hispanic, *n* (%)	4 (12.1%)	0 (0%)	.43
Age, years (IQR)	56 (48, 73)	70 (59.5, 78.8)	.02
Urgent care, *n* (%)	8 (24.2%)	1 (7.1%)	.34
Emergency care, *n* (%)	17 (51.5%)	6 (42.9%)	.75
Urine Collection, *n* (%)			.46
Catheterized	1 (3.0%)	1 (7.1%)	
Clean Catch	14 (42.4%)	8 (57.1%)	
Unspecified	18 (54.5%)	5 (35.7%)	
Colony forming units/ml, *n* (%)			.97
<50,000	8 (24.2%)	3 (21.4%)	
50,000–100,000	5 (15.2%)	2 (14.3%)	
>100,000	20 (60.6%)	9 (64.3%)	
Organism methicillin susceptibility, *n* (%)			
Susceptible, *n* (%)	28 (84.8%)	10 (71.4%)	.51
Urinary symptoms, *n* (%)	16 (53.3%)	9 (64.3%)	.722
Diabetes mellitus, *n* (%)	13 (39.4%)	6 (42.9%)	>.99
Intravenous drug use, *n* (%)	4 (12.1%)	0 (0%)	.43
Long term facility, *n* (%)	0 (0%)	3 (21.4%)	.04
Dialysis, *n* (%)	1 (3%)	1 (7.1%)	>.99
Urologic procedures, *n* (%)	6 (18.2%)	4 (28.6%)	.69
Hardware, *n* (%)	3 (9.1%)	2 (14.3%)	.99
Renal transplant, *n* (%)	2 (6.1%)	0 (0%)	.88

**Table 4.** Outcomes for Staphylococcus aureus bacteremia by intervention period

Outcome	Pre (*n* = 33)	Post (*n* = 14)	*P*-value
Follow- up blood culture performed, *n* (%)	16 (48.5%)	11 (78.6%)	.11
Early *S aureus* bacteremia, *n* (%)	12 (36.4%)	11 (78.6%)	.02
Late *S aureus* bacteremia, *n* (%)	21 (63.6%)	3 (21.4%)	.02
Deep seated infection (epidural abscess, vertebral osteomyelitis, endocarditis, or presumed endocarditis)	16 (48.5%)	0 (0%)	.004
Epidural abscess, *n* (%)	6 (18.2%)	0 (0%)	.22
Vertebral osteomyelitis, *n* (%)	6 (18.2%)	0 (0%)	.22
Endocarditis, *n* (%)	3 (9.1%)	0 (0%)	.61
Complicated urinary tract infection, *n* (%)	7 (21.2%)	7 (50%)	.10
Prostatitis, *n* (%)	3 (9.1%)	4 (28.6%)	.21
Central line infection, *n* (%)	2 (6.1%)	1 (7.1%)	>.99
Other site of infection, *n* (%)	4 (12.1%)	2 (14.3%)	>.99
Prolonged bacteremia without source, *n* (%)	1 (3.0%)	0 (0%)	>.99
*S aureus* bacteremia identified at another system prior to bacteriuria, *n* (%)	1 (4%)	0 (0%)	>.99
Mortality within 90 days, *n* (%)	4 (12.1%)	3 (21.4%)	.71

## Discussion

We implemented a microbiology alert prompting FUBC in all patients with SABU. The microbiology alert increased the rate of FUBC by 45% and increased the rate of early SAB detection from .6 to 2.0%. However, the intervention resulted in a 47% relative increase in ED revisits. Although the intervention increased FUBC in both males and females, early SAB was detected almost exclusively in males. The alert resulted in increased health care use with modest benefit to our patients. In our cohort, 11 to 15 FUBC were required to identify one early SAB. A risk-targeted microbiology alert may preserve the benefit to patients without unnecessarily increasing ED visits in lowest-risk populations.

We observed a 2% incidence of SAB within 90 days which is much lower than other cohorts.^2,4^ This is because we intentionally excluded patients who had blood cultures drawn with urine cultures upon initial evaluation and instead focused the intervention on patients presenting with first case SABU in ambulatory settings. This population poses more diagnostic uncertainty to clinicians and antimicrobial stewardship teams. Even after the intervention about 80% of patients did not have FUBC suggesting clinicians were using some criteria to risk stratify patients for the likelihood of SAB. Our findings suggest that routinely ordering FUBC in all patients with SABU is low yield if clinicians are already appropriately obtaining blood cultures in patients with systemic signs and symptoms of invasive disease on initial presentation. In this low-risk population, a high percentage of SABU cases likely represented colonization/contamination or were primary urinary tract infections that responded appropriately to antibiotic treatment.

An increase in ED revisits was observed with our intervention. Our intent was to increase FUBC through laboratory collection, however, we suspect that the electronic alert prompted repeat ED visit for both FUBCs and clinical examination. Although this may have been beneficial for a small number of patients, few of the ED visits required hospitalization, evidenced by similar early readmission rates between time periods. Better messaging regarding location of FUBC collection and a list of standardized questions to risk stratify patients may have prevented this increase in ED visits.

The rates of FUBC increased in both male and female patients, however early SAB was detected almost exclusively in males. Previous studies have identified male sex and advancing age as risk factors for SAB in the setting of SABU.^2,7^ Given the younger age of detection, easier contamination of clean catch specimens, higher risk of UTI, and *S. aureus* being a transient colonizer of the perineum in female patients, detection of SABU is more likely to reflect contamination and/or urinary tract infection in females than in males. It is possible that SABU in males may be a sign of unrecognized acute bacterial prostatitis which requires longer durations of therapy than UTI. Given these differences, FUBC for SABU may be more appropriate in relatively well-appearing males than females; additional research is needed to clarify which patient factors are most predictive of SAB complicating SABU. While awaiting risk-based prediction tools, we have modified our alert to read “*S aureus* is an uncommon urine pathogen and may be indicative of contamination or spread from invasive infection. Evaluate for signs of invasive disease including prostate infection.”

We were surprised that significantly fewer deep-seated SAB infections (epidural abscesses, vertebral abscesses and endocarditis, diagnosed within 3 mo of SABU) were observed during the postintervention time-period. Although this may reflect low sample size, the difference was significant. We were expecting earlier identification of these clinical entities. Biologically it is plausible that aggressive treatment of early SAB associated with urinary sources and/or prostatitis could decrease subsequent deep-seated infections.^9^ Prostate infections are difficult to diagnose, particularly in patients with immunocompromising conditions such as diabetes and may be a source for future septic emboli at distant sites.^10–12^ Alternatively, it is also possible that this observation was related to population differences over time (eg, lower number of females and intravenous drug use with SAB), increased identification of uncomplicated urinary tract infections with transient SAB, or better identification of deep-seated infections at initial evaluation during which concomitant blood and urine culture collection were performed. Future studies evaluating the impact of FUBC particularly in male patients should consider evaluating the impact of early SAB identification and treatment and the risk of deep-seated infections with and without SAB.

Our study has several limitations. First, our study is limited to a single health system. Patients may have sought care at facilities outside of the health-system in which the study was performed; however, this rate was likely similar between time periods. Second, changes in ED blood culture practices could have a dramatic impact on the importance of FUBC obtainment in patients with SABU. In our study, 50% of patients with SABU who had same day blood and urine culture collection were found to have SAB. Interventions to reduce overuse of blood cultures may shift the importance of FUBC in patients with SABU. Third, almost half of patients in both time periods received a prescription for empiric antibiotics which may have sterilized FUBC. FUBC may still be indicated as persistent SAB in the presence of therapy is associated with increased mortality^13^ and may indicate disseminated disease or abscesses amenable to earlier source control.^14^

## Conclusion

In a low-risk, low incidence population in which bacteremia was not initially suspected, an electronic, clinician-targeted alert linked to positive urine culture results effectively increased FUBC in patients with SABU by 45%, but the overall rate of FUBC remained low. The intervention resulted in earlier detection of bacteremia and increased ED visits. Early SAB was detected far more frequently in males. Risk-targeted strategies are needed to target appropriate use of follow-up blood cultures for patients with *S. aureus* bacteriuria who are at highest risk for bacteremia.

## Statistical analysis

Jared Olson, Brandon Webb.

## Supervision

Dustin Waters.
